# Wide-Awake Local Anesthesia No Tourniquet for Rhizarthrosis Surgery: Technique and Experience for 16 Consecutive Cases

**DOI:** 10.7759/cureus.45705

**Published:** 2023-09-21

**Authors:** Filipe Castelo, Cláudia Santos, Bárbara Costa, Ricardo Sousa, Raquel Ricardo, Pedro Batista, Daniel Ribeiro

**Affiliations:** 1 Orthopaedics and Trauma, Centro Hospitalar Universitário Cova da Beira, Covilhã, PRT

**Keywords:** basilar thumb arthritis, trapeziometacarpal joint, rhizarthrosis, walant, wide awake, orthopedics, hand surgery

## Abstract

Since it was described, wide-awake local anaesthesia no tourniquet (WALANT) has gained popularity. Our department has started using WALANT for hand surgery with increasing complexity. We present our results with WALANT rhizarthrosis surgery, including prosthetic replacement, trapeziectomy with suture button suspensionplasty and revision surgery. A retrospective review of all rhizarthrosis procedures under WALANT was performed from April 2021 to July 2022. We included patients who fulfilled inclusion criteria and had adequate imaging and clinical follow-up. A satisfaction survey was performed by telephone. Surgical time, complications, conversion to conventional anesthesia, pain, anxiety and global satisfaction were recorded. Tumescent anesthesia is performed 20-25 minutes before surgery, and is performed in four or five strategic locations that allow adequate anesthesia and vasoconstriction for the procedure to be comfortably carried out. We observed a series of 16 sequential surgeries involving 14 patients. All were female with a mean age of 65 years. Fourteen cases were performed due to primary rhizarthrosis, eight trapeziectomies with suture button suspensionplasty, six prosthetic replacements, and two revision surgeries. One patient needed to be converted to conventional anaesthesia due to anxiety during the procedure. Mean procedure time was 73 minutes. There were no WALANT-related complications. Mean patient-reported satisfaction with the anesthetic technique was a 9 (on a scale from 1 to 10) and 100% of patients would choose to undergo surgery with WALANT anesthesia for a future procedure. We find it useful to actively engage the patients during surgery to keep them comfortable and also help the surgeons assess stability and functional results. After wound closure, the hand is shown to the patient and he performs various tasks. There is somewhat of a learning curve for rhizarthrosis surgery under WALANT; patient comfort can be achieved through an adequate anesthetic technique and reassurance before and during surgery. We recommend that the first few cases be done in the presence of an anesthesiologist and a fasting patient in case there is a need to convert to conventional anesthesia. Wide awake rhizarthrosis surgery, even revision surgery, is safe and pain-free. Patient-reported satisfaction is also high. The authors find that including patient participation in their own surgery might be promising for post-op rehabilitation. There are limitations in this study such as the absence of a control conventional anesthesia group, the satisfaction questionnaire was not done immediately post-operatively, as such, a memory bias cannot be excluded, and it is not yet validated for the Portuguese population.

## Introduction

Protocols for the use of wide-awake local anaesthesia no tourniquet (WALANT) in upper limb surgery have expanded and gained ground in recent years. Initially described by Lalonde as an anesthetic alternative for repairing flexor tendon injuries of the fingers, nowadays it is used for procedures of increasing complexity in the upper limb [1-7].

The severe acute respiratory syndrome coronavirus 2 (SARS-CoV-2) pandemic was a driving event for WALANT surgery due to the restrictions on infrastructure and human resources that were felt. The adoption of WALANT anesthetic techniques allowed us to maintain ambulatory surgical activity during the pandemic period. Due to its advantages, some centers, including ours, continued this practice after the end of pandemic restrictions [8,9].

The advantages mentioned in WALANT surgery can be divided into three categories: patient-related, surgeon-related or health service-related. The advantages related to the patient are the absence of a motor block, adequate anesthesia, exemption from sedation or tourniquet, exemption from pre-operative exams, or suspension of anti-aggregation or hypocoagulant medication. Regarding advantages for the surgeon, adrenaline-induced hemostasis provides adequate visualization, interaction with the patient during the procedure allows for the initiation of postoperative care teaching, and the absence of motor block allows for the assessment of robustness and stability of different techniques through active mobilization. For health services, the advantages focus on reducing the occupancy of ward beds, operating rooms, costs per procedure, human resources, and hospital waste [10-12].

One of the most complex diseases treated under WALANT anesthesia is rhizarthrosis. Lalonde described his anesthetic technique for isolated trapeziectomy in 2011, and Müller published the first case description of a trapeziometacarpal (TMC) arthroplasty with prosthesis under WALANT in 2018 [3,7].

## Materials and methods

Patient selection

We retrospectively reviewed all consecutive rhizarthrosis surgeries under WALANT between April 2021 and July 2022; these were performed in a single orthopedic department and by a single surgeon. Patients were contacted by telephone and completed a modified satisfaction questionnaire based on that of Davison and Lalonde. The survey was carried out by a surgeon other than the main surgeon, at least one year post-operatively. This questionnaire is not validated for the Portuguese population and was adapted for the Portuguese language by the surgical team. The stage of rhizarthrosis was defined using the Eaton-Littler classification [13,14].

Inclusion criteria

We included all patients undergoing surgical treatment for rhizarthrosis under WALANT who had at least one year of follow-up, surgical description, outpatient follow-up, preoperative and postoperative imaging studies. We did not exclude patients who needed conversion to conventional anesthesia, as this was one of the end-points for this study.

Statistical analysis

This was a descriptive retrospective study and no comparative statistical analysis was performed.

WALANT technique

The anesthetic solution used was similar to that described by Lalonde: 6ml of 2% lidocaine (120mg), 0.2ml of 1mg/1ml epinephrine (200ug), 2ml of 8.4% sodium bicarbonate (168mg) diluted in 14ml of saline solution 0.9% making a total of 22ml of solution per syringe. The concentrations of this solution are: 0.55% lidocaine, 0.76% bicarbonate, and epinephrine at a dilution of 1:100,000. We use 22 ml syringes for user comfort with 25G needles. Depending on the procedure performed, we use two (44ml) to three (66ml) syringes of anesthetic solution. The maximum dose of lidocaine (when mixed with epinephrine) is 7mg/kg. This value is reached in patients weighing less than 50kg who require three syringes of solution to carry out the procedure, in these cases we use 1% lidocaine in our solution. The surgical incision is drawn with a skin marker and skin disinfection is carried out with a chlorhexidine solution. After tumescent anesthesia, we wait 20 to 25 minutes for the anesthetic and vasoconstrictive effect to be maximum [10,15].

**Figure 1:**
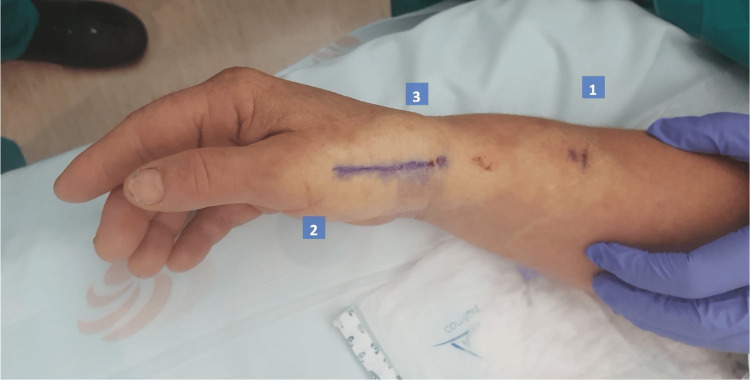
Anesthesia injection sites 1 to 3 1 - anatomic superficial radial nerve block 2 - Incision anesthesia 3 - Intra-articular anesthesia

**Figure 2:**
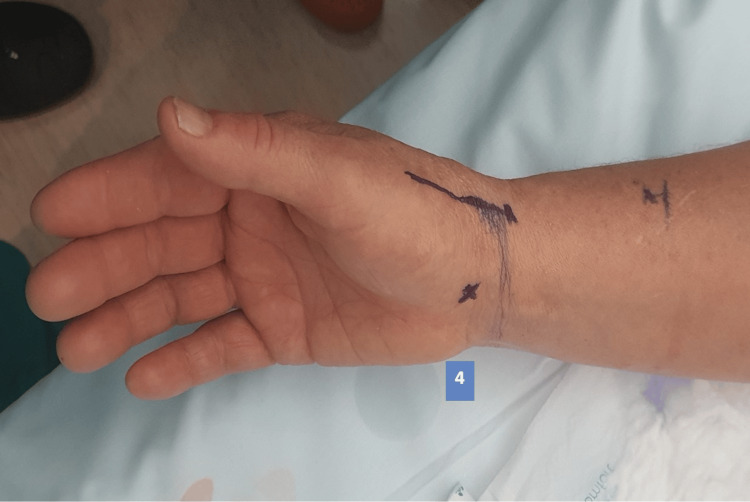
Anesthesia injection site 4 4 - scaphoid tubercle

**Figure 3:**
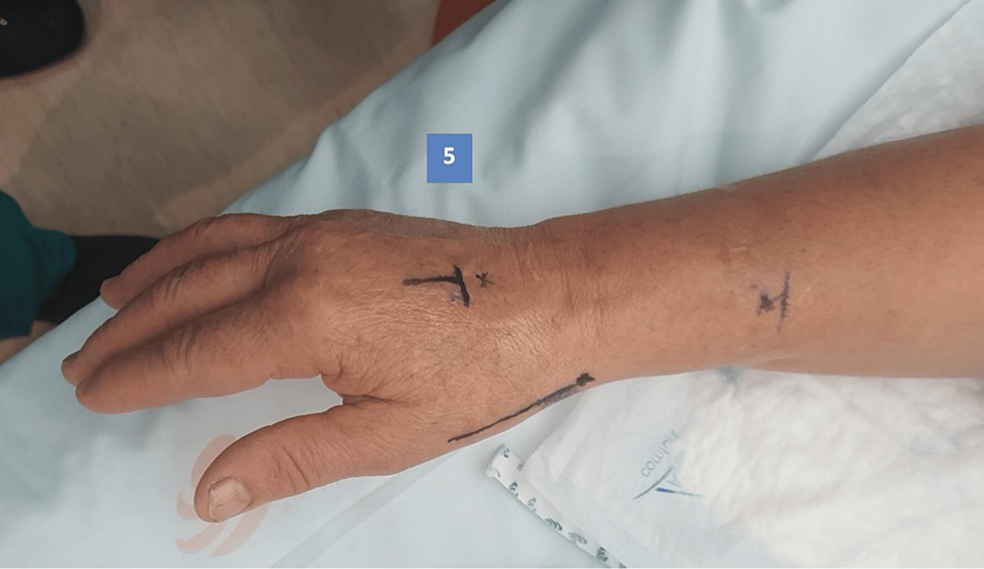
Anesthesia injection site 5 5 - base of second metacarpal and web-space

The injection sites to obtain adequate anesthesia depend on the procedure to be performed; in the case of simple trapeziectomy or implant arthroplasty, four sites are anesthetized. When we perform trapeziectomy with suture button suspensionplasty, it is necessary to anesthetize five areas. The first is a nerve block of the peripheral radial nerve, the second is the surgical approach (dorso-radial), then the TMC joint is infiltrated, the fourth site is the proximal tubercle of the scaphoid (limits pain in the vicinity of the scaphotrapeziotrapezoid joint). When performing a suture button suspensionplasty, the fifth site to be anesthetized is the base of the second metacarpal, including the periosteum, first and second web-space. Anesthetic injection sites are illustrated in Figures 1-3. Volume of solution to inject for each site is described in Table 1.

**Table 1:** Anesthetic injection volume per site

Injection site	Solution volume
1 – Radial nerve block	10ml
2 - Incision	22ml
3 – Intra-articular	2-4ml
4 – Scaphoid tubercle	8-10ml
5 – Second metacarpal and web-space	22ml

## Results

We identified 16 surgeries that met inclusion criteria. These were performed on 14 patients. All were female with an average age of 65 years (53-81). We performed eight trapeziectomies with suture button suspensionplasty (Microlink^TM^ System; Conmed Corporation, Largo, FL, USA), six arthroplasties with implant (Maïa^TM^ dual mobility; Groupe Lépine, Genay, France) and two revision surgeries. One revision surgery was an implant removal, trapeziectomy and suture button suspensionplasty, due to stiffness of the first web-space. The other was the excision of a volar osteophyte from the base of the first metacarpal due to postoperative conflict in a patient submitted to a prosthetic replacement. Eaton-Littler stages for rhizarthrosis varied between 2 and 4 and 50% of cases were stage 3.

All patients underwent surgery on an outpatient basis and all were discharged on the same day. The first four cases were operated while fasting and with the presence of an anesthesiologist in case there was a need to convert to conventional anesthesia. We had an average operative time of 73 minutes (60-101). We performed three types of accessory procedures: retrograde release of the first compartment of the extensor tendons, stabilization of the first metacarpophalangeal joint (MCPJ) with a Kirschner wire and reinsertion of the volar plate of the MCPJ with an anchor (in cases with MCPJ instability). There were three postoperative complications that required revision surgery. We did not identify any WALANT-related complications, but there was a need to convert the second patient to conventional anesthesia (patient anxiety). The recorded complications that required revision surgery were a conflict due to volar osteophyte of the first metacarpal, contracture of the first web-space, and prosthetic instability. The first two were reviewed under WALANT anesthesia, the last under conventional anesthesia. A detailed population description and results are available in Table 2.

**Table 2:** Population description N: case number; E-L: Eaton-Littler; CA: conventional Anesthesia; F: female; NA: not applicable; PR: prosthetic replacement; TSS: trapeziectomy and suture suspension; RS: revision surgery; MCPJ: metacarpophalangeal joint; NR: not recorded Patient age is represented in years. Surgical time is represented in minutes.

N	Age (years)	Sex	E-L Stage	Procedure	Accessory procedure	Conversion to CA	Complications	Surgical Time (minutes)
1	58	F	3	PR	No	No	Osteophyte impingement	71
2	61	F	2	PR	No	Yes	No	90
3	72	F	3	PR	No	No	No	97
4	62	F	3	PR	No	No	No	NR
5	74	F	3	PR	No	No	First web-space rigidity	NR
6	54	F	4	PR	No	No	Prosthetic Instability	101
7	57	F	2	TSS	No	No	No	70
8	69	F	4	TSS	No	No	No	60
9	77	F	4	TSS	1^st^ compartment release	No	No	NR
10	53	F	3	TSS	MCPJ pinning	No	No	NR
11	81	F	3	TSS	No	No	No	68
12	63	F	3	TSS	1^st^ compartment release and volar plate repair	No	No	NR
13	67	F	4	TSS	No	No	No	NR
14	67	F	4	TSS	Volar plate repair	No	No	92
15	74	F	NA	RS	No	No	No	NR
16	61	F	NA	RS	No	No	No	NR

**Figure 4:**
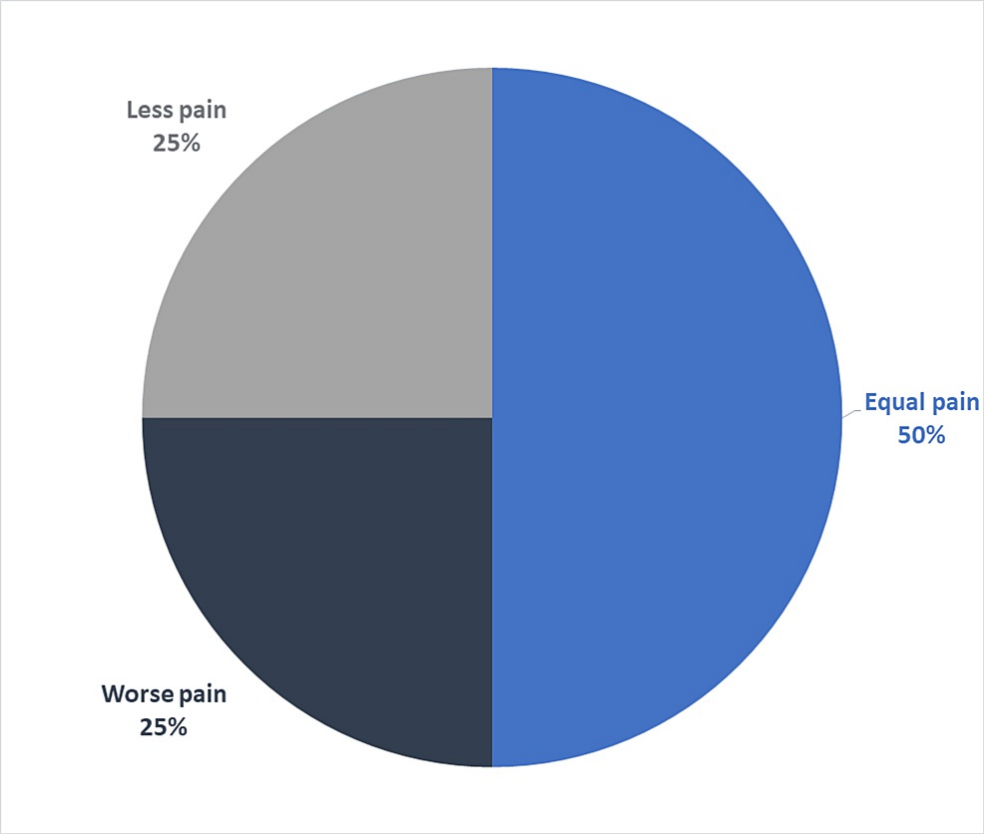
Anesthesia needle pain when compared with a blood analysis needle (in percentage of total patient-reported scores).

**Figure 5:**
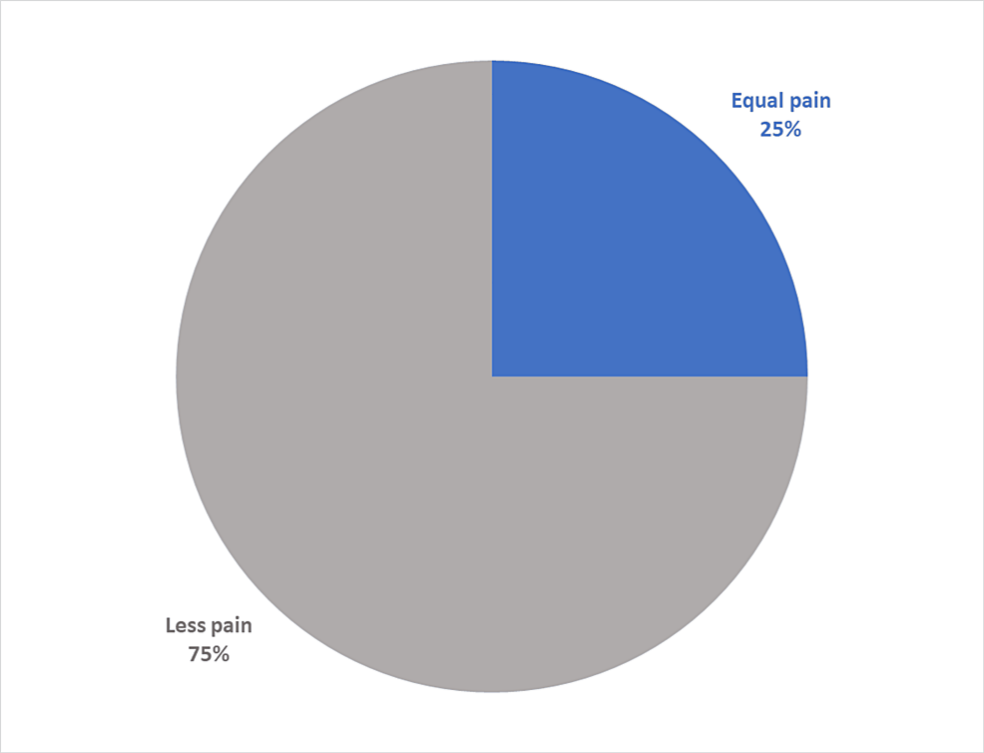
Pain during surgery when compared with a routine dental procedure (in percentage of total patient-reported scores).

A satisfaction questionnaire with the anesthetic technique was carried out on 12 patients (two were not able to adequately answer the questions). The questionnaire was adapted from the one created by Lalonde. We can highlight that 100% of patients would be willing to undergo surgery again with WALANT anesthesia. Seventy-five percent of patients compare the pain of the initial anesthesia sting as equal to or milder than a blood test. The average pain during the procedure was 2 (1 = no pain and 10 = maximum pain) and 100% reported that, during the surgery, the pain felt was the same or milder than during a dental procedure (Figures 4, 5). Detailed results of the questionnaire can be found in Table 3 [13].

**Table 3:** Patient-reported satisfaction Pain scale was defined from 1 to 10 (1 = no pain and 10 = extreme pain). Anxiety scale was defined from 1 to 10 (1 = not anxious and 10 = extremely anxious). Satisfaction scale was defined from 1 to 10 (1 = not satisfied and 10 = very satisfied). Values are represented as mean (±SD) of total patient-reported scores. SD: Standard deviation

Evaluated variable	Mean(±SD) patient-reported score (1 to 10)
Needle pain	2.50 (±1.57)
Pain during surgery	2.25 (±1.96)
Pain after surgery (before hospital discharge)	1.92 (±1.56)
Anxiety before anesthesia	4.33 (±3.94)
Anxiety during anesthesia	2.42 (±1.98)
Anxiety during surgery	2.25 (±1.91)
Anxiety after surgery (before hospital discharge)	2.00 (±2.00)
Global satisfaction	9.42 (±0.99)

## Discussion

There were no WALANT-related complications such as extremity ischemia, lidocaine or adrenaline toxicity. Our complication rate is in accordance with existing literature for rhizarthrosis surgery under conventional anesthesia [16-18]

Patient interaction and having the opportunity to witness the results of their surgery immediately after is of added value for their rehabilitation. With a painless thumb, it is possible to teach and show the range of mobility achieved immediately after surgery (Video 1). We recently started to have a physiotherapist present in the operating room, in order to provide the first teachings on the day of surgery. Since there is no motor block, we can actively test the stability and tension applied in an environment closer to reality. The benefit that dynamic tests can bring has yet to be proven for rhizarthrosis surgery, however, a reduction in prosthetic dislocation rates and more adequate tensioning in suspensionplasty or ligamentoplasty techniques are advocated. An additional benefit is reducing the surgeon's exposure to ionizing radiation, as the patient can position their hand autonomously to acquire images with the C-arm [19].

**Video 1:** Wide awake rhizarthrosis suture-button suspensionplasty intra-op patient education.

The surgical time recorded was within the values described by some authors, however this data is little explored in the available literature and many articles do not mention it. Thorkildsen et al. (2019) reported mean operating times of 96 minutes for trapeziectomy with tendon interposition and 75 minutes for implant arthroplasty. Yao et al. (2017) registered a surgical time of 93 minutes for trapeziectomy and suture button suspension. Weiss et al. (2019) and Gray and Meals (2007) report shorter operating times, but advocate simpler procedures like trapeziectomy with suture suspension or trapeziectomy with temporary Kirschner wire fixation. Although a comparative study was not carried out, it is not the authors' experience that surgical time is longer under WALANT than under conventional anesthesia. The waiting time of 20-25 minutes after anesthesia also seems to be comparable to that spent using conventional anesthetic techniques. Some authors advocate shorter waiting times for anesthesia and vasoconstriction in minor procedures [18,20-24].

There is, however, a learning curve for performing these techniques under WALANT, especially if the surgeon is unfamiliar with this anesthetic technique. Our recommendation is that the first cases be performed with a fasting patient and in the presence of an anesthesiologist, in case it is necessary to convert to conventional anesthesia (either for reasons related to the patient or the surgeon) [25].

In the vast majority of cases, the anxiety that the patient may present before or during the procedure can be controlled by the surgeon with words of comfort and explanation of what will be done, and what they can expect to hear and feel during the procedure (demonstrate sensation and sound of power tools comparing it to a trip to the dentist for example). Our satisfaction questionnaire supports these data, since patients report feeling less anxious during the procedure than before it began. In our experience, the use of anxiolytics is harmful to the procedure and the patient's experience, as it reduces their ability to cooperate during the procedure. Not requiring the use of a tourniquet is also beneficial for the patient's comfort as they do not experience the associated discomfort [26].

The average overall satisfaction with the anesthetic technique was 9 (on a scale of 1 to 10, with 1 being “not at all satisfied” and 10 being “very satisfied”). One hundred percent of patients would be willing to undergo surgery again under WALANT. This finding was previously published by Larsen and Hansen (2021) when he interviewed 50 patients undergoing implant arthroplasty under WALANT [22].

To the authors' knowledge, there is no literature to date involving the use of WALANT for revision surgeries. The two patients undergoing revision under WALANT were also satisfied with the anesthetic technique performed.

This study has some limitations; it is a retrospective study without a comparison group, the satisfaction survey is not validated for the Portuguese population and was adapted by the surgical team. This survey was also performed at least one year after the procedure, as such a memory bias cannot be excluded. The study also includes a small sample size which may not be representative for larger-sized populations. Although the objective of this paper is to demonstrate the feasibility of rhizarthrosis surgeries under WALANT, the fact that different procedures were included can be detrimental.

## Conclusions

WALANT rhizarthrosis surgery is safe, provides adequate anesthesia, and is comfortable for patients. This is reflected in a high satisfaction rate with this anesthesia. The rate of surgical complications is comparable to available literature and no WALANT-related complications occurred. The protocol used by our team is adapted from that described by Lalonde for isolated trapeziectomy. The changes proposed allow for more complex procedures including revision surgery. Due to the existing learning curve, we recommend the presence of an anesthesiologist during the first cases, to safeguard for the possibility of conversion to conventional anesthesia. A memory bias cannot be excluded from this retrospectively reviewed series of cases; the satisfaction survey was carried out at least one year after surgery and has not yet been validated for the Portuguese population. Prospective studies with larger populations and conventional anesthesia control groups are necessary to reach more robust conclusions.
